# Exosome as potential biomarkers for gastrointestinal tumors

**DOI:** 10.1097/MD.0000000000024509

**Published:** 2021-02-12

**Authors:** Jinlong Zhang, Shudan Fu, Wenshuo Chen, Haijin Chen

**Affiliations:** Zhujiang Hospital of Southern Medical University.

**Keywords:** diagnostic significance, exosomes, gastrointestinal tumors, meta-analysis, prognostic significance

## Abstract

**Introduction:**

Exosomes are polyvesicles that are formed by invagination of intracellular lysosomal particles, and are released into the extracellular matrix after the fusion of polyvesicular outer membrane and cell membrane. In the body, immune response, antigen presentation, cell migration, cell differentiation and tumor invasion are closely related to tumorigenesis and tumor progression. This study aimed to conduct a meta-analysis for evaluating the clinicopathological, diagnostic and prognostic significance of exosomal expression in gastrointestinal tumors.

**Methods:**

The original English articles were systematically searched in the online databases. The diagnostic accuracy, prognostic utility and clinicopathological correlation of gastrointestinal tumors were investigated. The quality assessment for studies of diagnostic accuracy II and Newcastle-Ottawa scale were used for quality evaluation, and the data was strictly extracted to judge the deviation of the study.

**Results:**

A total of 14 studies with 1837 gastrointestinal tumor patients were included. The change in exosomal expression showed significant correlation with poor clinicopathological parameters (tumor diameter: combined *P* = .00024394; differentiation: combined *P* = 2.796e-08; lymphatic metastasis: *P* = 9.610e-07; distant metastasis: combined *P* = .00017326; pathological classification: combined *P* = .00875213; invasion depth: combined *P* = 3.504e-08) carcinoembryonic antigen (combined *P* = . 04458857) and tumor location (combined *P* = .00145983). The difference in the area under the curve between gastrointestinal tumor patients and healthy people showed an area under the curve of 0.89 (95%Cl 0.85–0.91) and heterogeneity of 0.59, 95% CI=[0.55–0.68]. The sensitivity was 0.88 (95%Cl 0.83 mi 0.91), the specificity was 0.72 (95%Cl 0.63 mi 0.80), and the diagnostic odds ratio was 18 (10–33). The results of survival analysis revealed that the abnormally expressed exosomes were significantly correlated with poor overall survival (hazard ratio =2.81, 95% CI: 2.02–3.93, P=0.013^∗^ 62.7%^∗^).

**Conclusion:**

The abnormally expressed exosomes might act as auxiliary biomarkers in diagnosing gastrointestinal tumors and demonstrated good prognostic significance in predicting the survival of patients with gastrointestinal tumors.

## 1 Introduction

Gastrointestinal tumors are the leading cause of cancer-related morbidity and mortality worldwide, and the incidence of these tumors is increasing year by year.^[1]^ Among all malignant tumors, the incidence and mortality of colorectal cancer (CRC) ranked fourth and third in China.^[2]^ Patients with gastrointestinal tumors had a poor prognosis, but the tumors diagnosed in the early stage demonstrated a better prognosis.^[3]^ Routine blood biomarkers are not enough in diagnosing or predicting the prognosis of patients with gastrointestinal tumors. Therefore, the development of new diagnostic and prognostic biomarkers is imperative in reducing gastrointestinal tumor-related deaths.^[4]^

Exosomes are small membrane vesicles (30–150 nm) that contain complex RNA and proteins, and are considered as a new research perspective. Currently, exosomes are specifically referred to as discoid vesicles, and have a diameter of 40 to 100 nm.^[5]^ The exosomes were first seen in sheep reticulocytes in 1983 and was termed as “exosome” by Johnstone in 1987. Various cell types can secrete exosomes both in normal as well as pathological conditions. These mainly come from polyvesicles that are formed by invagination of intracellular lysosome particles, and are released into the extracellular matrix after the fusion of outer membrane of the polyvesicle and the cell membrane.^[6,7]^ In recent years, exosomes have been considered as biomarkers^[8–22]^ in predicting gastrointestinal malignant tumors, but these findings still remain controversial. Hence, in this study, the clinicopathological, diagnostic, and prognostic significance of exosomes in patients with gastrointestinal malignant tumors were summarized by conducting a meta-analysis.

## 2 Methods

### 2.1 Methods literature retrieval

This study was conducted in accordance with the Preferred Reporting Items for a Systematic Review and Meta-analysis of Diagnostic Test Accuracy Studies The PRISMA-DTA Statement published in 2018.^[23]^ Online databases such as PubMed, Wanfang data knowledge service platform and China National knowledge Infrastructure were searched for eligible studies and the studies that discussed the diagnosis, prognosis or clinicopathological significance of exosomes in gastrointestinal malignant tumors were retrieved. The following search terms in different combinations were used for searching in different databases: colorectal cancer, colon cancer, colorectal tumor, gastric cancer, exosome, clinicopathological features, clinicopathological features, clinicopathological parameters, diagnosis, sensitivity, specificity, area under curve, area under the curve (AUC), ROC curve, risk ratio, Overall survival (OS), hazard ratio (HR). The patients with gastrointestinal malignant tumors were considered as the case group, and those with benign lesions or healthy individuals were considered as the control group.

**The inclusion criteria were as follows**: studies

(1) that reported the diagnostic accuracy, prognostic utility or clinicopathological correlation of gastrointestinal malignant tumors;
(2) on gastrointestinal malignant tumors confirmed by histopathology; and
(3) that have sufficient data to draw 2X2 table, or available HR values and 95% confidence interval (CI), or available P values for clinicopathological correlation to study the clinical application of exosomes in gastrointestinal malignant tumors.

The exclusion criteria are as follows:

(1) reviews, basic research, meta-analysis, letters or case reports;
(2) poor quality of research studies; and
(3) studies from which the data cannot be extracted or secondary calculation is not consistent with that of the original text.

### 2.2 Data extraction

The eligibility of all the studies was evaluated and data such as

(1) baseline information (the first author's name, publication date, number of cases, control source, detection matrix, method, reference gene, demarcation point, exosome type, and expression),
(2) clinicopathological data of exosome expression and age, sex, tumor location, tumor diameter, differentiation, serous invasion, lymphatic metastasis, distant metastasis and TNM stage (*P* value),
(3) diagnostic data [sensitivity, specificity, AUC value, or true positive, false positive, false negative, true negative (TN) value]; and
(4) prognostic data ([follow-up time, HR value and 95%CI of OS) were extracted.

### 2.3 Quality assessment

The quality of research related to diagnosis was graded according to the diagnostic accuracy of study quality assessment II (QUADAS II) checklist,^[24]^ which included 7 questions on patient selection, indicator testing, reference criteria, procedure and time. The risk of bias is classified as “no,” “yes” or “unclear.” Only those questions with an answer “yes” were given a score of 1 point, otherwise no score was given. The relationship between study quality and outcome was assessed by the Newcastle-Ottawa scale (NOS),^[25]^ which assessed study selection, comparability, and risk of outcomes. The study with QUADAS II score of 4 stars and the NOS checklist score of 6 stars was considered to be of high quality.

### 2.4 Statistical analysis

STATA software (version 12.0) was used to analyze the clinicopathological and prognostic significance of exosomes in gastrointestinal tumors. The sensitivity, specificity, positive likelihood ratio, negative likelihood ratio and total diagnostic advantage were higher than the diagnostic odds ratio (DOR) and area under the ROC curve (AUC). Heterogeneity among the studies was assessed by X2 and I2 (I-square) tests, and the cut-off point was set as *P* < .05 in X2 test or *I*^2^ > 50%. The associations between exosome expression and clinicopathologic parameters were determined using the *P* values by combining with Fisher exact test.^[26]^ HR and 95%CI were combined based on multivariate Cox hazard regression analysis.^[27]^ The sensitivity and metaregression tests were used to identify the underlying causes of heterogeneity. Publication bias was quantitatively judged by Deeks’ funnel plot asymmetry test, Begg and Egger tests. *P* < .1 was considered as statistically significant difference.

## 3 Results

### 3.1 Search results

**Figure 1:**
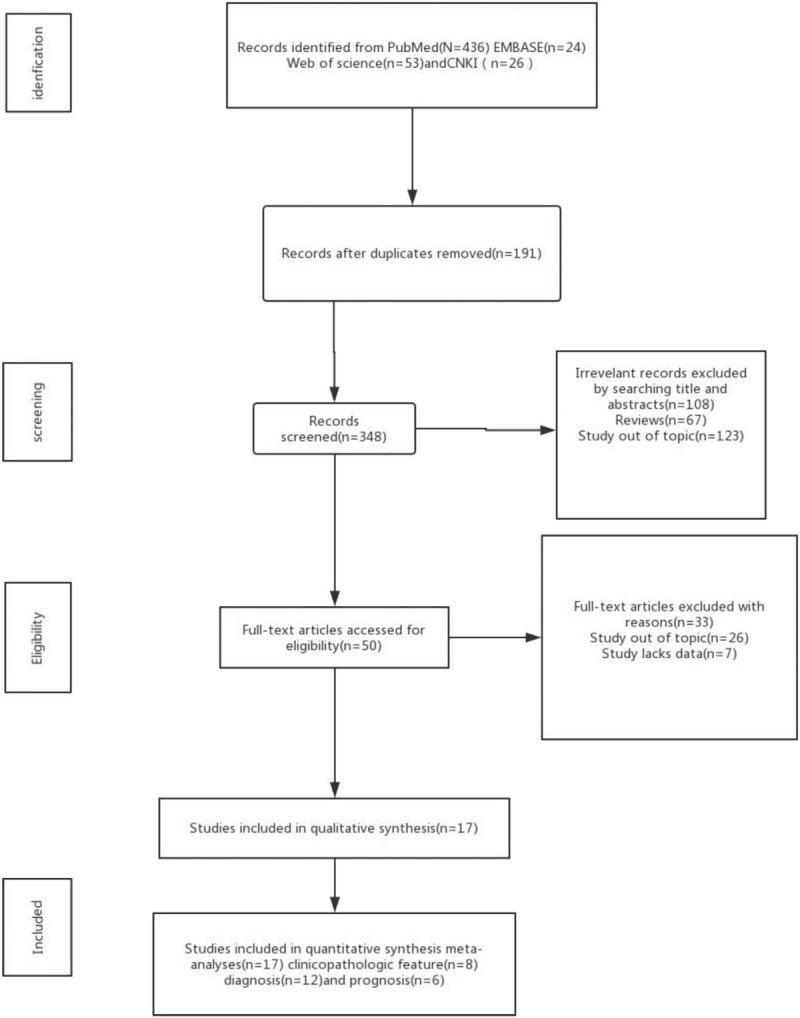
Flow chart of study selection process.

The study selection procedure was shown in Figure 1. In the initial search, a total of 439 publications from PubMed, EMBASE, Web of Science, SCOPUS, and Chinese National Knowledge Infrastructure databases that met the inclusion criteria were retrieved. Of these, 303 publications were identified as duplicates and so were eliminated. After reading the titles and abstracts, 120 records were eliminated as no association between circRNA expression and CRC was observed or others were review articles. Verification of full-texts of these excluded 16 articles as they were out of topic or lacked sufficient data. Finally, 13 studies were included in the quantitative meta-analysis.

### 3.2 Study characteristics and study quality

Of the 13 studies included, 8 studies summarized clinicopathologic parameters,^[9–11,13,14,16,18,19]^ 9 on diagnosis,^[9–14,16,18,19]^ and 7 on prognosis.^[10,12,14,15,17,20]^ The baseline characteristics of all included studies are summarized in Tables 1 and 2. All 13 studies were carried out in Asia. A total of 1430 CRC cases were included, and the sample size ranged from 32 to 318. All CRC cases were diagnosed by histological and pathological examinations. The tissue samples were obtained prior to clinical treatment. circRNA expression level was determined using quantitative real-time polymerase chain reaction (qRT-PCR) or RNA sequencing, and the reference genes included GAPDH,^[10–17,19,20]^ 18S rRNA,^[9]^ and U6.^[18]^ Six types of circRNAs were recognized as tumor promoters,^[12,15,16,18,20]^ and 7 as tumor suppressors.^[9–11,13,14,17,19]^ Survival analysis was available in 2 studies, and 3 articles contained data on HR and 95% CI, whereas the HR values in the remaining 4 articles were unclear and calculated indirectly.

**Table 1:** Main characteristics of the meta-analysis for diagnostic performance and clinicopathologic association of exosomes in patients with gastrointestinal tumors.

Study	Location	Tumor type	Patient number	Control number	Control type	Sample type	Exosomal signature	Expression status/Biological function	Method	Cutoff-value	Reference gene	AUC	95% CI	Incorporate CEA's AUC	Sensi Sensi Tivity	Spspecif, Specifciity
LI M 2019	China	Colorectal cancer	40	52	Paired noncancerous counterparts	plasma	Exosomal miR-92b	Down-regulated/Tumor-supressor	RT-qPCR/2–ΔΔCT		Cel-39-3q miRNA	0.793				
LIU T 2016	China	Colorectal cancer	148	10	Paired noncancerous counterparts	Serum	Exosomes lncRNA CRNDE-h	Up-regulated/ Tumor promotor	RT-qPCR	0.02	GAPDH	0.892	0.860–0.918	0.913	70.3%	94.4%
Zhao R 2018	China	Gastric cancer	126	120	Paired noncancerous counterparts	Serum	Exosomal HOTTIP	Up-regulated/ Tumor promotor	RT-qPCR/2–ΔΔCT	1.720	GAPDH/UBC	0.827	1.720		69.8%	85%
Zou SL 2019	China	Colorectal cancer	133	60	Paired noncancerous counterparts	Serum	Exosomal miR-150-5p	Down-regulated/Tumor-supressor	RT-qPCR/2 –ΔΔCT	median	Cel-miR-39	0.870		0.910	81.0%	76.1%
Wang J 2017	China	Colorectal cancer	50	50	Paired noncancerous counterparts	plasma	Exosomal miR-125a-3p	Up-regulated/ Tumor promotor	RT-qPCR/ΔCT=CTmiR−CTreference		GAPDH	0.684		0.8552		
Fu HL 2018	China	Gastric cancer	80	80	Paired noncancerous counterparts	Peripheral Blood	Exosomal TRIM3	Down-regulated/Tumor-supressor								
Wang N 2017	China	Gastric cancer	130	130	Paired noncancerous counterparts		Exosomal miR-19b-3p miR-106a-5p	Up-regulated/ Tumor promotor	qRT-PCR	1.5	Graph	0.7690.786	0.678–0.9480.659–0.926		95%95%	90%90%
LI J 2017	China	Colorectal cancer	102	80	Paired noncancerous counterparts	Peripheral Blood	GPC1 exosome	Up-regulated/ Tumor promotor			U6mRNA					
Mitsuo 2017	Japan	Colorectal Cancer	326	30	Paired noncancerous counterparts	plasma	Exosomal MicroRNA-21	Up-regulated/ Tumor promotor	qRT-PCR/2 –ΔΔC		GAPDH					
Yu HT 2017	China	Colorectal cancer	70	60	Paired noncancerous counterparts	Serum	ExosomalMiR. 21,ExosomaliR. 92a	Up-regulated/ Tumor promotorUp-regulated/ Tumor promotor	qRT-PCRqRT-PCR		RNAU6RNAU6	0.860.87	0.79–0.930.80–0.93		83.6%70.9%	81.4%86.4%
Zhang HL 2019	China	Gastric cancer	47	32	Paired noncancerous counterpart		ExosomalLncRPN 2–4	Down-regulated/Tumor-supressor	qRT-qPCR/2 –ΔΔCT	1.31	RNAU6	0.772	0.662–0.882		87.2%	59.4%
Li Y 2017	China	Gastric cancer	51	32	Paired noncancerous counterpart	Serum	Exosomal DANCR	Up-regulated/ Tumor promotor	qRT-qPCR/2 –ΔΔCT	2.50	RNAU6	0.777	0.678–0.876		68.6%	84.4%

**Table 2:** Main characteristics of meta-analysis on the prognosis of exosomes in patients with gastrointestinal tumors.

Study	Location	Exosomal signature	Follow-up time	HR	95% CI	*P*	n
Florian Oehme 2019	German	exosomal long non-coding RNA HOTTIP	80.4 mo	4.5	1.69–11.98	.0027	
MitsuoTsukamoto 2016	Japan	Exosomal miR-21	55 mo	2.28	1.81–5.74	<.01	
Yasunori Matsumoto 2016	Japan	Exosomal		3.15	1.11–11.41	.030	
Liu T 2016	China	Exosomal long noncoding RNA CRNDE-h	44.9 mo	2.724	1.530–4.849	.001	
Zhao R 2018	China	Exosomal long noncoding RNA HOTTIP	33 mo	1.63	1.19–2.23	.0022	246
Yuichiro Mik 2018	Japan	Exosomal CD63	unclear	3.29 (OR)	2.38–4.60)	<.0001	595
Zou S L 2019	China	exosomal miR-150–5p		4.52	2.37–6.90	.018	

CI = confidence interval, HR = hazard ratio.

Study bias and quality assessment by QUADAS II and NOS checklists are shown in Tables 3 and 4. The rating scores of all eligible studies for diagnosis ranged from 4 to 6, and for prognosis ranged from 6 to 8, which indicated high methodological quality of all the included studies.

**Table 3:**
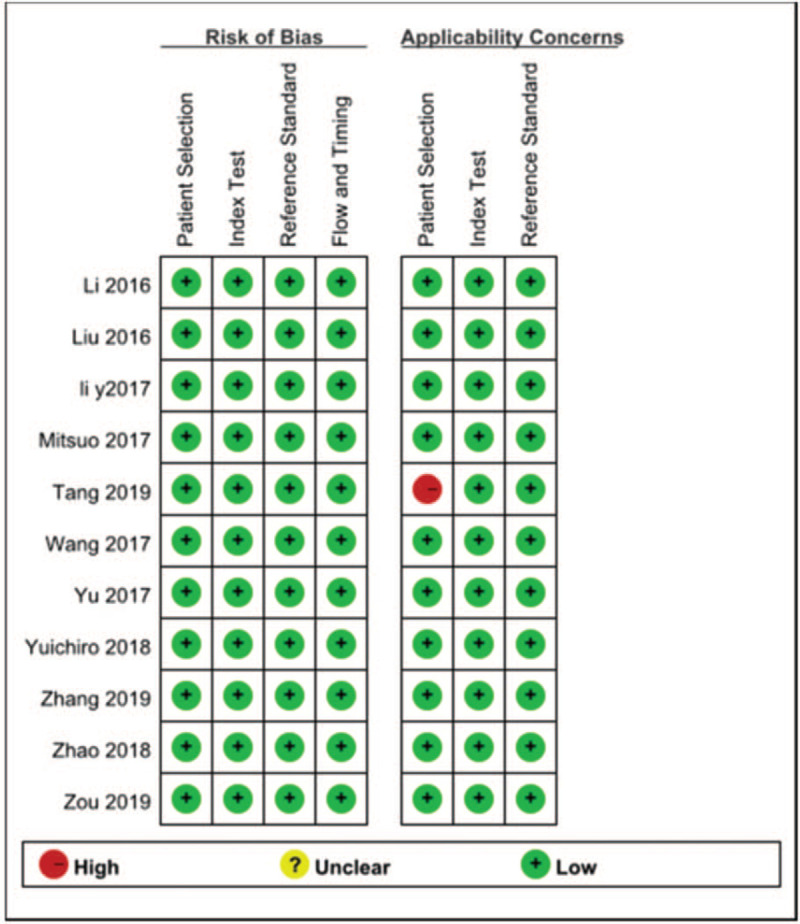
Study quality of diagnostic studies by QUADAS II checklist.

QUADAS = quality assessment for studies of diagnostic accuracy.

**Table 4:** Study quality and bias in the retrospective cohort studies judged by the Newcastle-Ottawa Scale (NOS) checklist.

		Cohort selection				Comparability	Outcome ascertainment		
Study	Total stars	Representativeness of the exposed cohort	Selection of the Non-exposed cohort	Ascertainment of exposure	Demonstration that outcome of Interest was not present at start of study	Comparability of cases and controls on the basis on the design or analysis	Assessment of outcome	Was followed up long enough for outcomes to occur	Adequacy of follow up of cohorts
Florian Oehme 2019	8	1	1	1	1	1	1	1	1
Liu T 2016	7	1	1	1	0	1	1	1	1
Zhao R 2018	8	1	1	1	1	1	1	1	1
Mitsuo Tsukamoto 2016	7	1	1	1	0	1	1	1	1
Zou SL 2019	5	1	1	1	0	1	1	0	0
Yuichiro Mik 2018	6	1	1	1	0	1	1	0	1

### 3.3 Meta-analysis of clinical parameters

The relationship between the exosomes and clinicopathological characteristics of gastrointestinal patients was shown in Table 5. The change in exosomal expression showed significant correlation with poor clinicopathological parameters (tumor diameter: combined *P* = .00024394; differentiation: combined *P* = 2.796e-08; lymphatic metastasis: *P* = 9.610e-07; distant metastasis: combined *P* = .00017326; pathological classification: combined *P* = .00875213; invasion depth: combined *P* = 3.504e-08) carcinoembryonic antigen (combined *P* = .04458857) and tumor location (combined *P* = .00145983). In contrast, there was no significant correlation between age (*P* = .1317845) and sex (*P* = .66845137).^[8]^

**Table 5:** Association between exosomal expression and clinicopathological features in gastrointestinal tumors by Fisher exact test.

Clinicopathological factors	Combined *P* value	*X*^2^ value	Enrolled studies
Age	.1317845	17.498599	6
Gender	.66845137	13.058832	8
Tumor location	.00145983	31.849308	6
Tumor differentiation	2.796e-08	59.489326	6
Local invasion	3.504e-08	58.950709	6
Regional lymph node metastasis	9.610e-07	50.923235	6
Distant metastasis	.00017326	22.317508	2
Tumor size	.00024394	29.647607	4
Carcinoembryogenic antigen	.04458857	15.849302	4
Pathological type	.00875213	26.623065	6

### 3.4 Diagnostic performance

**Figure 2:**
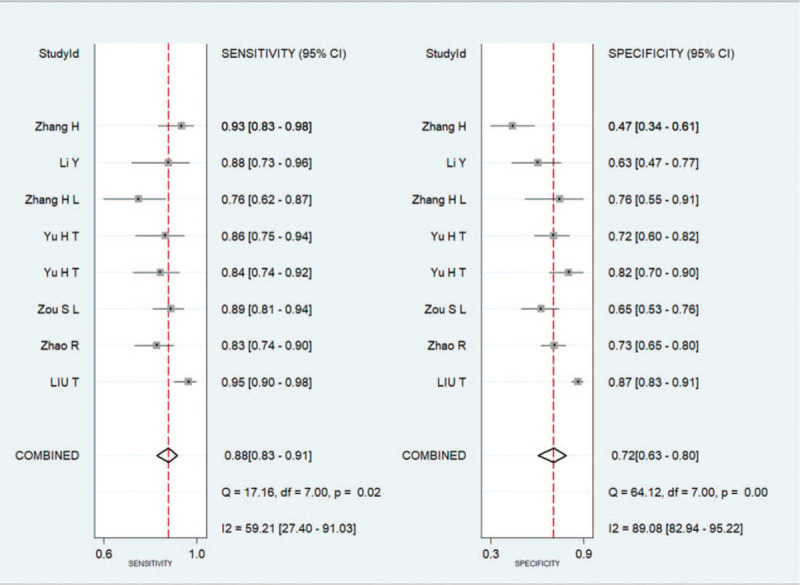
Forest plots of the combined sensitivity and specificity.

**Figure 3:**
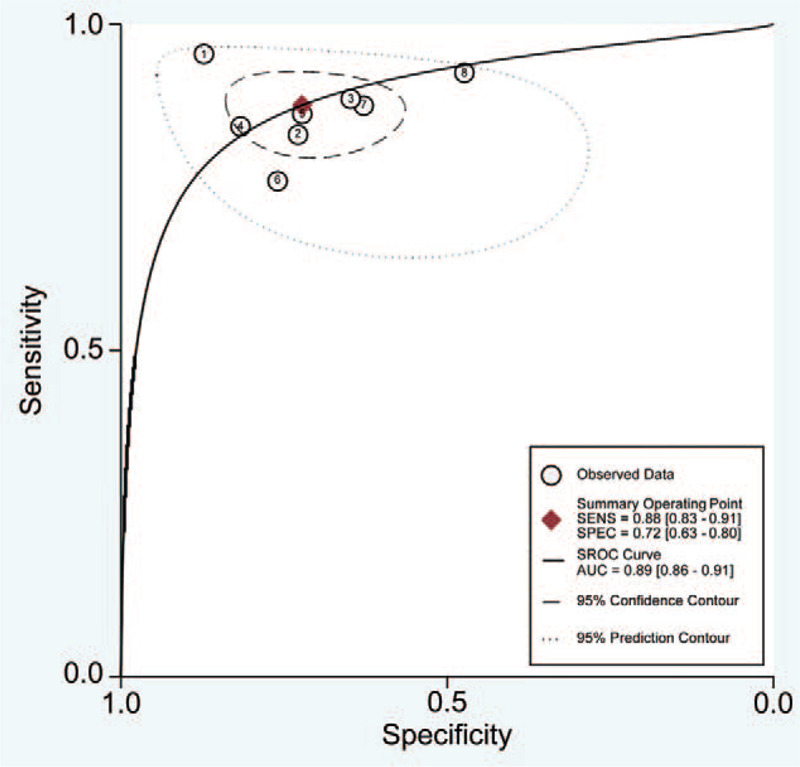
SROC Curve and AUC for exosomes expression in diagnosing gastrointestinal tumors.

The diagnostic parameters of exosomes for differentiating gastrointestinal tumors from non-tumor controls were as follows: 0.88 (95%Cl 0.83 mi 0.91), the specificity was 0.72 (95%Cl 0.63–0.80), (Fig. 2) DOR 18 (10–33), SROC curve AUC 0.89 (95%Cl 0.86–0.91) (Fig. 3) heterogeneity 0.59, 95% CI = [0.55–0.68]

### 3.5 Publication performance

**Figure 4:**
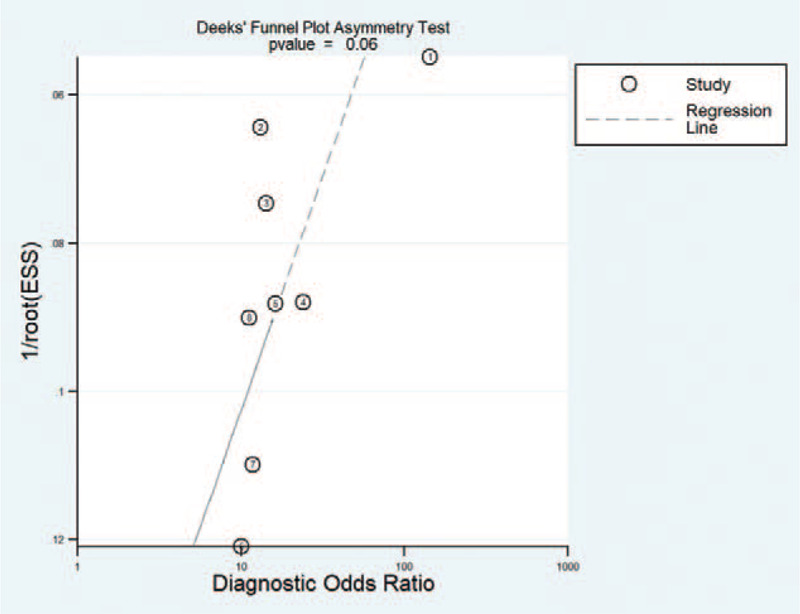
Publication bias assessed by the Deek's funnel plot for the overall diagnostic effect.

Deek funnel plot asymmetry test showed that no evidence of publication bias (*P* = .06) in diagnostic analyses (Fig. 4). Therefore, the possibility of publication bias was excluded.

## 4 Discussion

Gastrointestinal tumors are the most important cause for cancer-related deaths worldwide. It is imperative to develop new diagnostic and prognostic biomarkers due to growing incidence of gastrointestinal tumors. Exosomes are widely used as biomarkers for diagnosis and prognosis of new cancer types, especially gastrointestinal tumors. However, there is no meta-analysis study on the abnormal expression of exosomes in gastrointestinal tumors. This study systematically analyzed the clinical, diagnostic and prognostic significance of abnormal expression of exosomes in gastrointestinal tumors.

Studies have shown a significant relationship between abnormal expression of exosomes and gastrointestinal tumors. This study found that the abnormally expressed exosomes are related to tumor diameter, differentiation, lymphatic metastasis, distant metastasis, TNM staging and depth of invasion, suggesting that the abnormally expressed exosomes are involved in the progression of gastrointestinal tumors. In contrast, no significant correlation with age and sex was observed.

ROC curve is a comprehensive index that reflects the sensitivity and specificity of continuous variables. Our summary results showed that the expression of exosomes demonstrated high diagnostic efficacy in gastrointestinal tumors, with a sensitivity of 0.88 and a specificity of 0.72. The combination of exosomes with AUC showed that the exosomal levels in 89% randomly selected CRC patients was lower or higher than that of the normal controls. The combined DOR also acts as an important indicator for formal meta-analysis of diagnostic test performance studies. In this study, the total DOR was 18 (higher than 1.0), which indicated that the imbalance in the exosomal expression acts as a powerful biomarker in diagnosing gastrointestinal tumors. As exosomes with different expression status might play different functions in gastrointestinal tumors, these can be used as new non-invasive biomarkers for the detection of gastrointestinal tumors.

**Figure 5:**
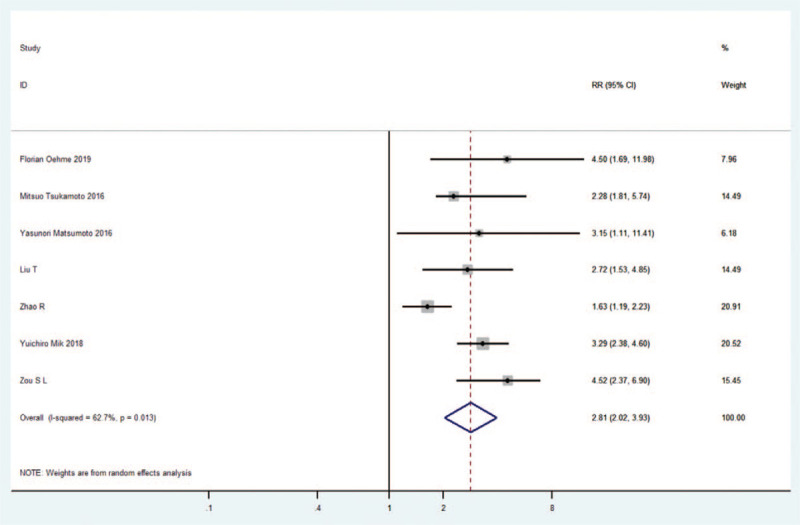
Forest plots of the combined HRs with 95%CIs respectively for the exosomes profiles in predicting the overall survival (OS) of patients with gastrointestinal tumors.

Studies have shown that the abnormal expression of exosomes has now become an independent risk factor for cancer OS. Consistent with these data, our combined effect size in CRC patients showed that the abnormal expression of carcinogenic exosomes demonstrated a close association with reduced OS time (HR = 2.81,95% CI: 2.02–3.93P = 0.013) (Fig. 5).

**Figure 6:**
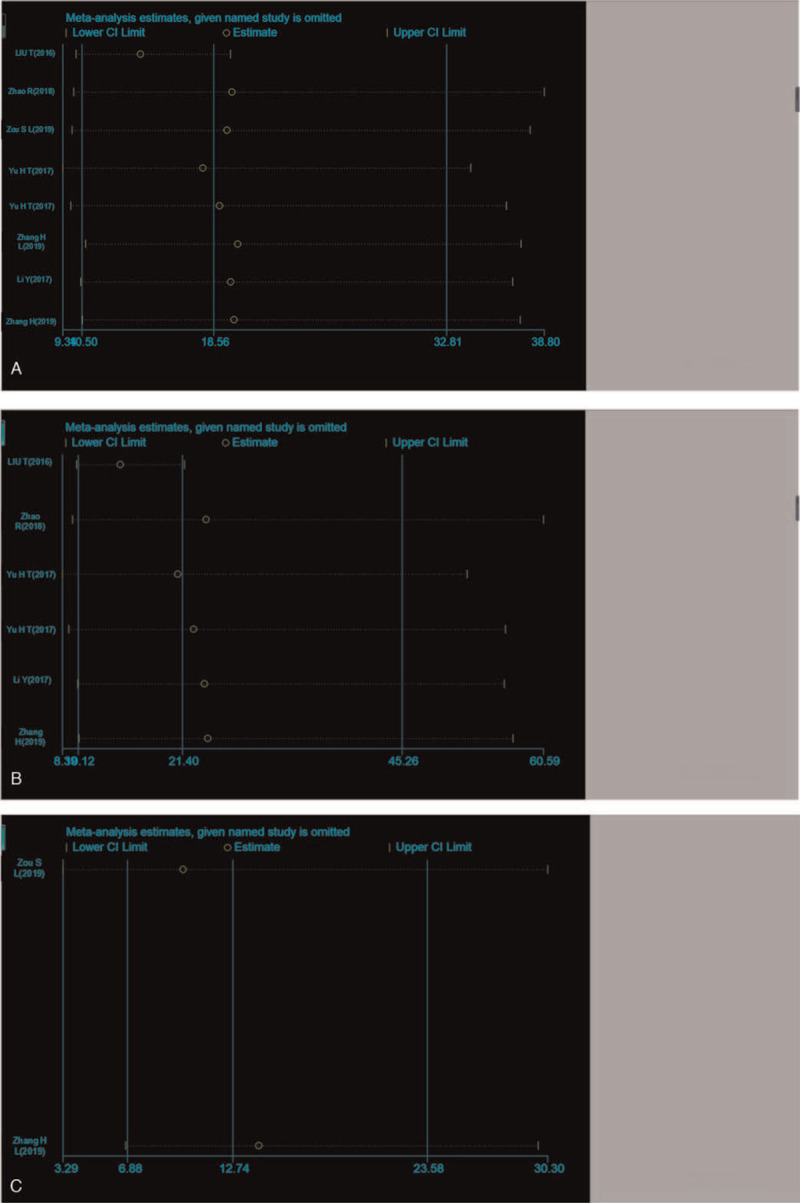
Sensitivity analysis of the outlier data for (A) the overall diagnostic studies, (B) the down-regulated exosomes profiles for diagnosis, as well as (C) the up-regulated.

To date, published studies have demonstrated that abnormal exosomal expression is associated with the diagnosis and prognosis of patients with gastrointestinal tumors. These findings are consistent with those observed in this meta-analysis. The sensitivity and specificity reported by Wang et al. were significantly higher than those reported by other studies. One explanation for this might be that compared with other studies (TNM stage I stage IV), the patients reported by Wang et al. had a more advanced TNM stage and a smaller sample size. The heterogeneity between the studies is mainly due to this difference. Another possible source of heterogeneity involves the quantitative analysis of exosomes. Thirteen types of exosomes with different expression status in gastrointestinal tumors were included, and quantitative analysis was based on different reference genes (GAPDH, 18S rRNA, Cel-miR-39 or U6); and therefore, the heterogeneity was generated in the pooled effects. On the other hand, Deek's funnel plot asymmetry test showed no evidence of publication bias (P = 0.06) for diagnostic analyses, suggesting that all pooled effect sizes were reliable. Since exosomes with different expression states might play different functions in gastrointestinal tumors, and so a subgroup analysis was performed. Stratified analysis based on exosome expression status revealed that the exosomes act as tumor promoters of higher diagnostic efficacy than those exosomes that act as tumor suppressors (Fig. 6) and exosomes based on serum sources had higher diagnostic efficacy than exosomes based on plasma or peripheral blood sources. However, the sample size was reduced in the subgroup analysis, resulting in compromising the accuracy.

A meta-analysis^[27]^ study has confirmed that high Circular RNA expression was associated with the diagnosis and prognosis of patients with CRC. These findings are consistent with the results observed in the present meta-analysis. The abnormal expression of exosomes showed association with the diagnosis and prognosis of patients with gastrointestinal tumors, but more studies with larger sample size were included. The diagnosis of patients with colorectal cancer revealed that merging with CEA can obviously improve the effect of AUC, improving the diagnosis. So, a more detailed analysis was conducted.

Moreover 2 studies^[28,29]^ showed that high CRNDE expression is associated with the progression of TNM and N stages in solid tumor patients, which is also consistent with the conclusion of our meta-analysis. The high expression of CRNDE often indicates poor prognosis, which is not exactly the same as our results. More studies were included and found that exosomes with low expression indicated a poor prognosis, providing a new idea for new therapeutic targets and monitoring indicators.

However, our study has some limitations. Firstly, not all the studies adopted blinding method, leading to a source of bias. Secondly, the HR values that are indirectly extracted might increase the insufficiency of statistical power. Thirdly, the impact of time variable on outcomes was neglected as the final follow-up intervals were different in the selected studies. Furthermore, several studies included a smaller sample size, affecting the accuracy of our pooled results. Well-designed studies with larger sample size are required for further study. Finally, population bias might exist in our analyses as most of the studies were conducted in China.

## 5 Conclusion

In summary, the results of this meta-analysis demonstrated that exosomes act as promising biomarkers in diagnosis and prognosis of patients with gastrointestinal tumors, and might be used as therapeutic targets.^[30]^ Further prospective studies on more types of exosomes are warranted in the future. By testing the exosomes that have the advantage of in non-invasive detection, clinicians could diagnose gastrointestinal tumors, and help patients with gastrointestinal tumors in predicting their prognosis.

## Author contributions

**Conceptualization:** Jin long Zhang, Wenshuo Chen, Haijin Chen.

**Data curation:** Jin long Zhang.

**Formal analysis:** Jin long Zhang, Haijin Chen.

**Investigation:** Jin long Zhang, Shudan Fu, Haijin Chen.

**Methodology:** Jin long Zhang, Shudan Fu, Haijin Chen.

**Project administration:** Jin long Zhang.

**Resources:** Jin long Zhang.

**Software:** Jin long Zhang, Shudan Fu, Wenshuo Chen.

**Supervision:** Jin long Zhang, Shudan Fu, Wenshuo Chen.

**Validation:** Wenshuo Chen.

**Writing – original draft:** Jin long Zhang, Wenshuo Chen.

**Writing – review and editing:** Jin long Zhang, Wenshuo Chen, Haijin Chen.

## Abbreviations

Abbreviations: AUC = area under the curve, CRC = colorectal cancer, DOR = diagnostic odds ratio, HR = hazard ratio, NOS = Newcastle-Ottawa Scale, OS = Overall survival, QUADAS = quality assessment for studies of diagnostic accuracy.
